# A mouse informatics platform for phenotypic and translational discovery

**DOI:** 10.1007/s00335-015-9599-2

**Published:** 2015-08-28

**Authors:** Natalie Ring, Terrence F. Meehan, Andrew Blake, James Brown, Chao-Kung Chen, Nathalie Conte, Armida Di Fenza, Tanja Fiegel, Neil Horner, Julius O. B. Jacobsen, Natasha Karp, Thomas Lawson, Jeremy C. Mason, Peter Matthews, Hugh Morgan, Mike Relac, Luis Santos, Damian Smedley, Duncan Sneddon, Alice Pengelly, Ilinca Tudose, Jonathan W. G. Warren, Henrik Westerberg, Gagarine Yaikhom, Helen Parkinson, Ann-Marie Mallon

**Affiliations:** MRC Mammalian Genetics Unit, MRC Harwell, Harwell Science and Innovation Campus, Harwell, OX11 0RD UK; European Molecular Biology Laboratory, European Bioinformatics Institute, Wellcome Trust Genome Campus, Hinxton, Cambridge CB10 1SD UK; Wellcome Trust Sanger Institute, Wellcome Trust Genome Campus, Hinxton, Cambridge, CB10 1SA UK

## Abstract

The International Mouse Phenotyping Consortium (IMPC) is providing the world’s first functional catalogue of a mammalian genome by characterising a knockout mouse strain for every gene. A robust and highly structured informatics platform has been developed to systematically collate, analyse and disseminate the data produced by the IMPC. As the first phase of the project, in which 5000 new knockout strains are being broadly phenotyped, nears completion, the informatics platform is extending and adapting to support the increasing volume and complexity of the data produced as well as addressing a large volume of users and emerging user groups. An intuitive interface helps researchers explore IMPC data by giving overviews and the ability to find and visualise data that support a phenotype assertion. Dedicated disease pages allow researchers to find new mouse models of human diseases, and novel viewers provide high-resolution images of embryonic and adult dysmorphologies. With each monthly release, the informatics platform will continue to evolve to support the increased data volume and to maintain its position as the primary route of access to IMPC data and as an invaluable resource for clinical and non-clinical researchers.

## Introduction

The International Mouse Phenotyping Consortium (IMPC) comprises the majority of the world’s largest mouse research centres, working together to generate and characterise a knockout mouse strain for 20,000 protein coding genes (Brown and Moore 2012a, b). The IMPC aims to ascribe function to every gene by measuring the physiological and morphological effects that result when a gene is inactivated. It builds upon resources from a number of previous projects including the mutant embryonic stem (ES) cells produced by the International Knockout Mouse Consortium (IKMC) (Ringwald et al. 2011; Bradley et al. 2012) and by previous, smaller scale high-throughput phenotyping projects such as the Eumorphia and Eumodic projects (Morgan et al. 2010). The IMPC is a publicly funded resource with all data freely available at www.mousephenotype.org.

To date, the IMPC has captured phenotype data for over 2000 mouse genes (1406 post-QC 600 in pre-QC) and data submissions are growing rapidly to achieve the goal of having 5000 strains phenotyped by the end of 2016. Production and phenotyping pipelines are established at each centre and data deposition is automated (see Table 1). Critical to the success of this project is a centralised comprehensive informatics infrastructure that captures, stores, manages and integrates all data produced. The Mouse Phenotyping Informatics Infrastructure Consortium (MPI2), a collaborative effort between the European Molecular Biological Laboratories- European Bioinformatics Institute (EMBL-EBI), Medical Research Council Harwell, and the Wellcome Trust Sanger Institute (WTSI), develops the IT infrastructure, databases, analysis software and web portals required to efficiently capture, manage, annotate, integrate and disseminate the data produced by the IMPC. The original MPI2 infrastructure (Mallon et al. 2012; Koscielny et al. 2014) has developed substantially to deal with emerging datatypes and it now supports the reproducibility of IMPC results by following the ARRIVE guidelines (Karp et al. 2015), has developed an online quality control and phenotypic comparison platform (Phenoview; Yaikhom et al. 2015) and provides versioned releases of a software package that performs automated statistical analysis of phenotype data (PhenStat; Kurbatova et al. 2015). A crucial feature of a successful informatics strategy is the ability to evolve as data are acquired and to support new user groups with intuitive and accessible tools. Here we present new features of the IMPC informatics platform developed to meet the needs of a growing number of diverse users while addressing increases in data volume and complexity (Table 2).

**Table 1:** The names and locations of the ten phenotyping centres which have thus far submitted data for the IMPC

Centre name	Centre location
Baylor College of Medicine	USA
Helmholtz Zentrum Munchen	Germany
MRC Harwell	UK
Institut Clinique de la Souris	France
The Jackson Laboratory	USA
The Toronto Centre for Phenogenomics	Canada
Nanjing University	China
RIKEN Tsukuba Institute, BioResource Center	Japan
University of California, Davis	USA
Wellcome Trust Sanger Institute	UK

**Table 2:** Summary of IMPC data releases to date, showing the number of lines with any data included in the release

Release	Date	Number of lines
1.0	16-Jun-14	301
1.1	26-Jun-14	484
2.0	06-Nov-14	535
3.0	06-Feb-15	1528
3.1	29-Apr-15	1540

### Rapid release of robust data

The IMPC is generating an ever-increasing volume of phenotype data. Seven male and seven female mice are analysed from 9 weeks of age until 16 weeks of age using a broad-based phenotyping pipeline that assesses every major biological system. As one-third of knockout mouse strains are expected to be embryonic lethal, a separate pipeline assesses embryonic dysmorphologies in these strains. A centralised database of IMPC agreed standard operating procedures (SOPs), IMPReSS (www.mousephenotype.org/impress), ensures that all phenotyping data and metadata are collected in a reproducible and standardised manner. Currently, data are successfully collected for over 1200 parameters represented in 24 adult SOPS. A critical component of the SOPs is the mapping of phenotypes to data parameters. For example, the glucose parameter in the clinical chemistry SOP is associated to “decreased circulating glucose” and “increased circulating glucose” terms. The IMPC uses the Mammalian Phenotype ontology (MP) developed by Mouse Genome Informatics to represent its phenotypes (Smith and Eppig 2009), which provides a robust classification of phenotypes and facilitates data integration with MGI (www.informatics.jax.org). When a mutant strain is determined to be a phenodeviant by being a statistical outlier for a parameter, the SOP ontology associations are used to assign a phenotype to the strain.

**Fig. 1:**
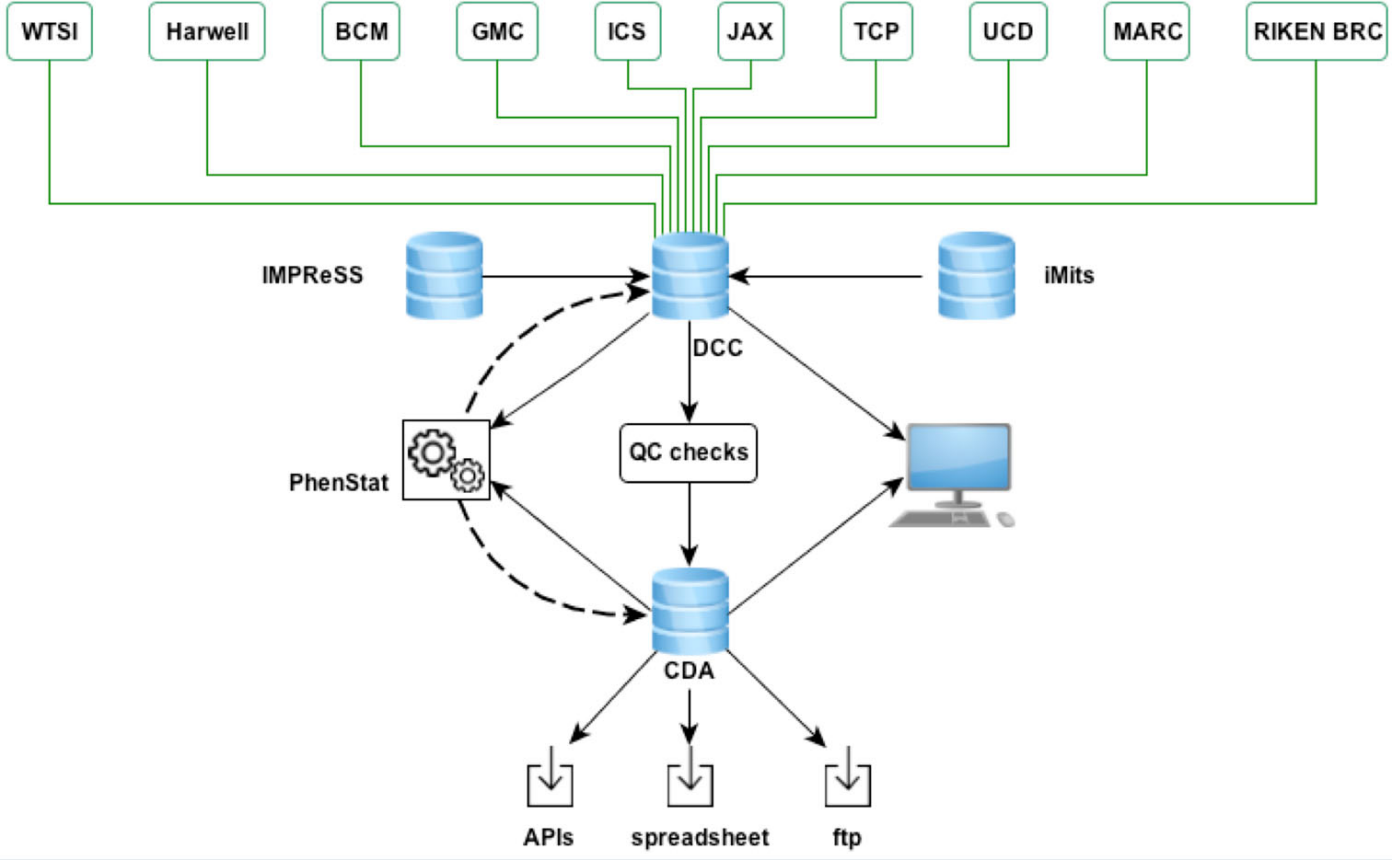
Data flow in the IMPC. The ten IMPC phenotyping centres upload data for collection and curation centrally at the DCC, followed by final statistical analysis and storage at the CDA for public dissemination and usage. Preliminary (pre-QC) data are viewable by external users immediately. Production and phenotyping processes are standardised and recorded by the use of the International Mouse Injection Tracking System (iMits) and International Mouse Phenotyping Resource of Standardised Screens (IMPReSS)

A robust data capture and release process is available to handle this large and complex data flow. Community feedback established that any release process should allow for swift user access to all information while ensuring any final results integrated with other resources is adequately quality-controlled (QC’d) and analysed. A data release strategy has, therefore, been implemented by MPI2 to meet these needs. Figure 1 illustrates the flow and release of data within the IMPC. Phenotype data are first generated and stored locally at the IMPC centres according to the standardised phenotyping pipeline. These data are then organised into specimen and experimental data files that are exposed to the project’s Data Coordination Centre (DCC) on a daily basis, guaranteeing regular updates to the centralised databases. Submitted data are validated against the live IMPC mouse production tracker database, iMits (www.mousephenotype.org/imits) and are immediately viewable by submitting centres on the PhenoView platform so they can view the status of their data and to allow reporting of data flow across all centres. Reporting tools inform on the success of the data upload, providing cumulative and monthly totals of specimens, procedures, and lines available. The data are then subjected to a preliminary statistical analysis (Kurbatova et al. 2015), associated with phenotypes and presented on the mousephenotype.org portal with “Pre-QC” labels on dedicated gene and phenotype pages, with all pre-QC data clearly visible by use of orange colour coding. Once data submitted to the DCC have passed automated validation steps such as verification of units, it undergoes a manual QC process by data wranglers who review outliers in the data and resolve any issues with the phenotyping centres. This manual process has proven to be critical in detecting anomalies in data and ensuring that the highest quality data are released for public usage. The wranglers also contribute for improved automation of QC processes, thus improving the data upload efficiency and release.

All data are periodically reviewed by the data wranglers to ascertain which sets of post-QC data are “complete”, i.e. have the minimum required number of animals as defined in the SOPs. When a dataset is both complete and QC-passed, a complete statistical analysis is performed at the project’s Core Data Archive (CDA) and final, robust, phenotype calls are made visible on the portal and distributed to other resources. These data are shown with the pre-QC data on the IMPC portal, for instances, where the centre has not finished phenotyping all the specimens, or has not completed all the tests. The result of this entire process is that users are able to access preliminary phenodeviant associations made within days of data submission as well as having robust associations made from QC complete data. The data release process is versioned, robust and reproducible to allow tracking of data between releases (www.mousephenotype.org/data/release).

### Enhanced search

**Fig. 2:**
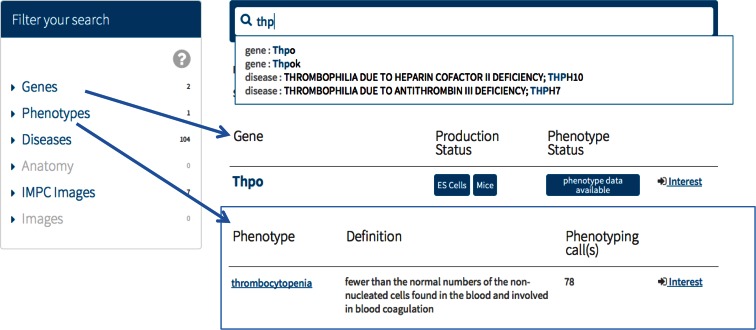
The IMPC search interface. Users can search by gene, disease, phenotype and anatomy. Filters on the left of search show summaries of how many results appear in each category, and selecting a filter will return a results table for that category. Gene results include status of mouse production, availability of data and ability to register interest in a gene. Phenotype filtered results give definitions, the number of genes associated to the phenotype, and ability to register interest

Key to the success of the IMPC is ensuring that our users can access data and materials relevant to their research. All data are accessible via the project’s unified portal via gene, phenotype, anatomy and disease specific views onto the phenotypic data. Users can enter free text describing genes, parameters or phenotypes through an intuitive search interface found on the home page and on the dedicated “Search” page of the IMPC online portal (Fig. 2). Filters appear to the left of the search and provide summaries for each data type. For example, the search for “thpo” returns two genes, one phenotype, seven images and 104 diseases. A new auto-suggest feature guides users in finding biological entities stored in the IMPC resource and appears in the box below the search while text is being added.

Gene queries allow users to see the phenotyping status of the gene, with links to both newly generated and legacy phenotyping data, as well as an indication of whether ES cells or live mice are currently available. Users can also register interest by the “interest” link on the right end of a results row. Once their interest is registered, a user will be alerted when new data are available or a production status change has occurred so that mice data can be retrieved and mouse or ES cell clones ordered.

Selecting the phenotype filter returns a table of MP terms related to the user’s query (Fig. 2). The results include the MP definition and the number of genes associated to this phenotype. Researchers can now indicate their interest in a particular phenotype term via the search page or the dedicated phenotype page (discussed below), and will receive a periodic email update about new strains associated to that phenotype. As we have a translational user group familiar with clinical terms rather than an assay or a mouse description, new mappings between the Human Phenotype ontology and the MP have been added. For example, when a clinical researcher enters “short nose”, they will be directed to the MP term “short snout”. Users may also search for diseases described in the genetic disease resources OMIM, DeCIPHER and Orphanet returning a disease page (discussed below); anatomical terms which direct to pages containing lacZ reporter images; and for images categorised by data parameter or anatomy.

In addition to online searches, data can be accessed and downloaded via one of three routes: programmatic access via the application programming interfaces (API), directly from a portal graph, or through ftp access for bulk data downloads.

### Relating IMPC resources to human diseases

To support translational research, a major new feature of the IMPC portal is the representation of human genetic diseases. Dedicated pages are now available on the web portal representing diseases as described by a number of genetic disease resources: OMIM (www.omim.org), Orphanet (www.orpha.net) and DECIPHER (www.decipher.sanger.ac.uk). These pages use the PhenoDigm resource to provide summary disease descriptions with links back to the respective resources, and list potential mouse disease models for a given disease (see Smedley et al. this issue).

**Fig. 3:**
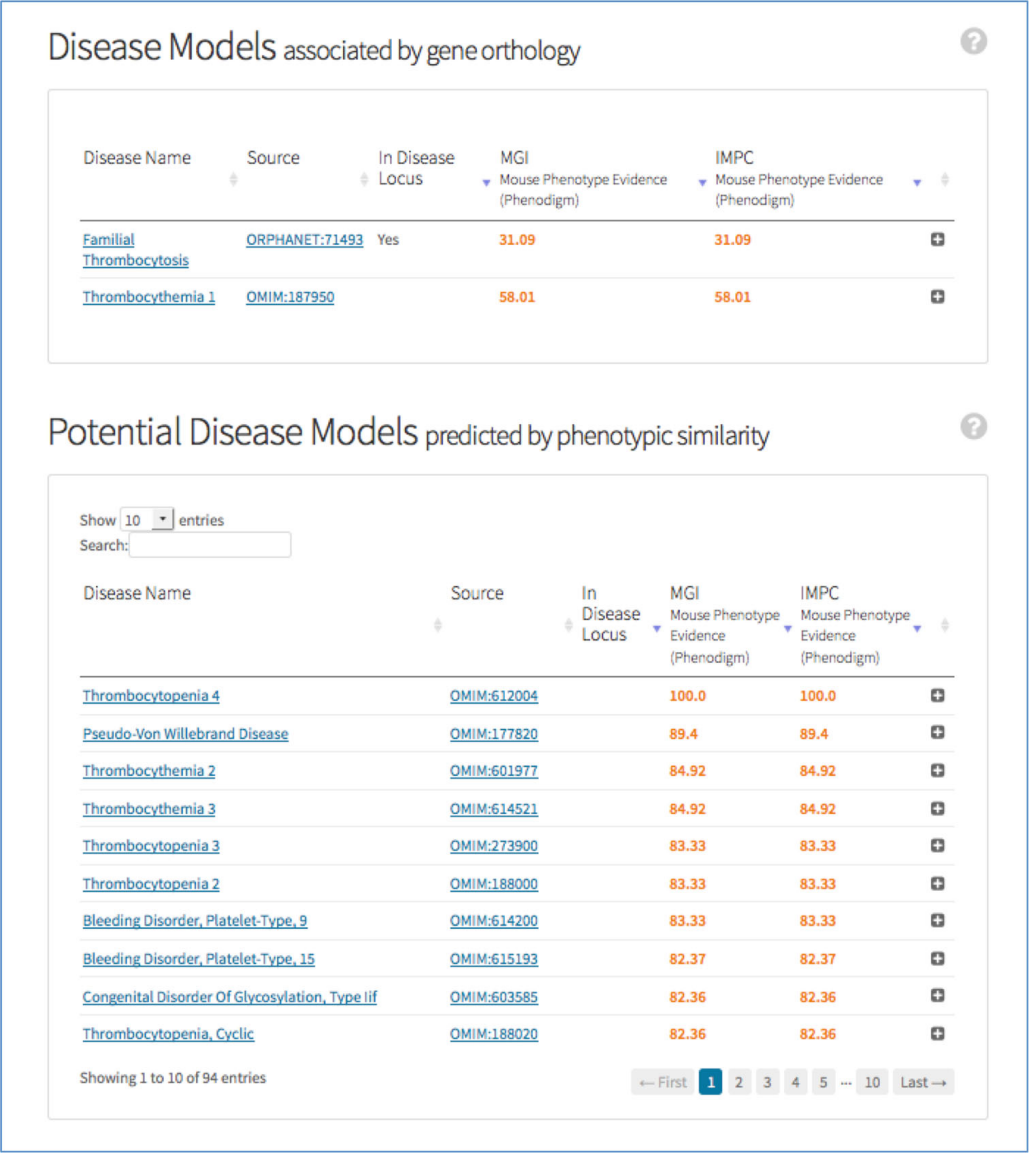
Diseases for which the gene *Thpo* is a potential model, as identified by determining if the gene is an orthologue of a human disease-causing gene (*top*) or by using the PhenoDigm to detect whether the phenotypes seen in IMPC *Thpo*-carrying mice overlap with any diseases (*bottom*)

Human diseases also appear on a gene page in two modules that reflect the two complementary methods used to associate a knockout mouse strain to a human disease (Fig. 3). The first method determines if a knockout gene is orthologous to a human gene associated to a disease by the DECIPHER, OMIM or Orphanet resources. These putative models are depicted on the portal as “Disease Models associated by gene orthology”. In Fig. 3, the top module represents how *Thpo* is orthologous to human genes implicated in Thrombocythemia 1 (OMIM: 187950) and Familial Thrombocytosis (ORPHANET: 71493). The second method is to use an automated disease-association method based on the OwlSim algorithm (Smedley et al. 2013), which detects IMPC mouse strains that have a strong phenotype overlap for a given disease. The advantage of this second method is that it finds models for human diseases where a genetic component is not known or not constrained to a single gene, often the case for rare genetic diseases. Figure 3 demonstrates how the significantly reduced blood platelet counts associated with *Thpo* knockout mice may be a viable model for other bleeding disorders such as Thrombocytopenia 4 (OMIM: 612004). This approach has been successfully used to guide clinical researchers to causative gene loci in human diseases (Smedley et al. this issue).

In the tables generated by both methods, a phenotype similarity score is provided from the PhenoDigm resource. This score, ranging from 0—no phenotype overlap to 100—perfect phenotype overlap, reflects and ranks phenotypic similarity between an IMPC mouse strain and human disease patients. This allows users to assess if the mouse strain is demonstrating important features of the human disease. Scores are also provided on MGI’s phenotype curation of other knockout mouse strains, as phenotypes described in the literature complement the broad-based phenotyping of the IMPC by being narrower and more specific (Fig. 3).

### New gene pages

Genes are currently the most highly searched category within the IMPC. As the number of genes with phenotype data grows, we have implemented gene-level summaries of the large volume of phenotype data collected. From the top of each gene page, users receive the essential information needed to identify a gene: name, symbol, related synonyms. Links are provided to the Ensembl genome browser, to MGI, and to human orthologs based on Ensembl’s Compara (Vilella et al. 2009) Links are also provided to register interest in the gene, and to ordering information for IMPC resources (discussed below).

**Fig. 4:**
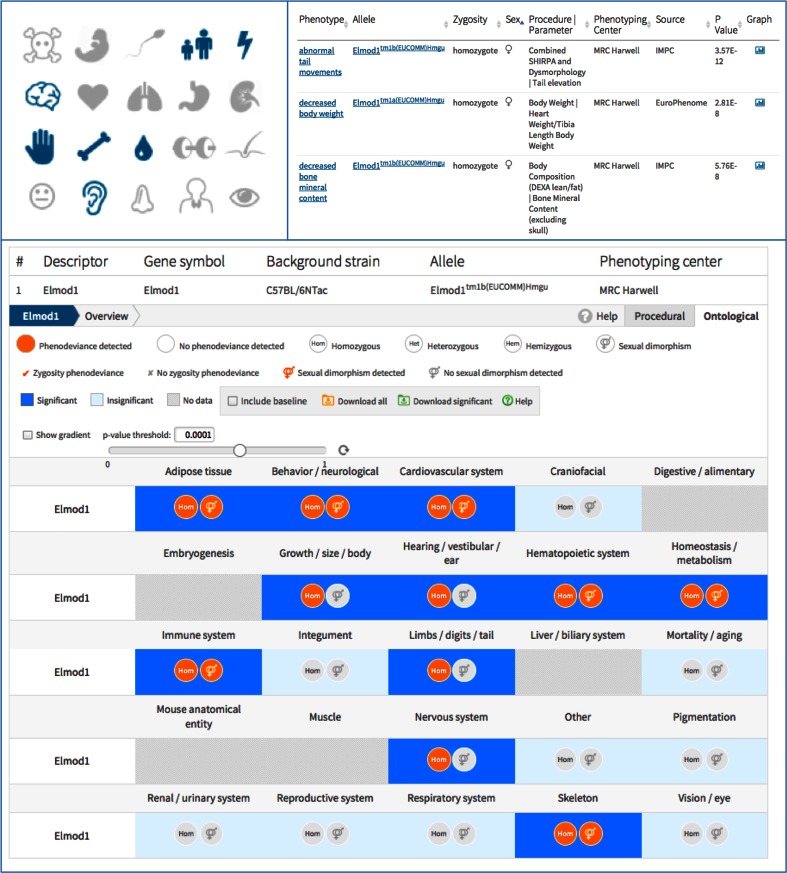
Text, Icon and Heatmap view for Elmod1 provide a rapid overview of the complex phenotypes and an integrated view on pre- and post-QC data (IMPC Data release 3.2)

Below the gene identification module, three views are provided for the associated phenotypes. Figure 4 illustrates the three complementary views (icon, table and heatmap) for Elmod1, a GTPase activator whose knockout mouse strains have a wide array of phenotypes. Text and icon phenotype overviews are presented at the top of the module to allow users to quickly assess if associated phenotypes are relevant to their research interests. A phenotype association table appears below the overview and lists the MP terms associated with knockout strains for this gene based on both pre- and post-QC phenotype data. A heatmap allows users to explore the data dynamically by changing significance thresholds for phenotype associations. Each phenotype call can be explored further via graphs illustrating mutant and control data for the parameter of interest. All data can be downloaded from the interface in spreadsheet format for offline use.

A defining goal of the IMPC is to provide easy access to mouse strains to the research community. A shopping cart icon on the top of each gene page navigates the user to an order section. This section contains descriptions of the mutant alleles and reporter gene elements, and provides links to repositories where strains or embryonic stem cells may be obtained. These repositories include the Knockout Mouse Project (KOMP) repository (Lloyd 2011) (www.komp.org), the European Mouse Mutant Archive (EMMA; Wilkinson et al. 2010) (www.infrafrontier.eu/) and the European Mouse Mutant Cell Repository (www.eummcr.org). Where strains have yet to be archived at a repository, contact information for an IMPC centre is provided so users may request strains.

### Sexual dimorphism

Early analysis of the IMPC’s data indicates that over 10 % of the genes tested show sexual dimorphism. All gene-phenotype associations are tested for sexual dimorphism in the IMPC statistical analysis and an icon is used to indicate if a phenotype is detected in one or both sexes. All data are analysed and graphed by sex as well as by genotype, and sex-specific effect sizes and p-values are provided in the analysis tables of each graph. For example, Elmod1 has sexually dimorphic calls such as abnormal tail movements only seen in females as visible on the heatmap (orange flag on sex symbols) and tables (Fig. 4).

### Identifying genes with shared phenotypes

**Fig. 5:**
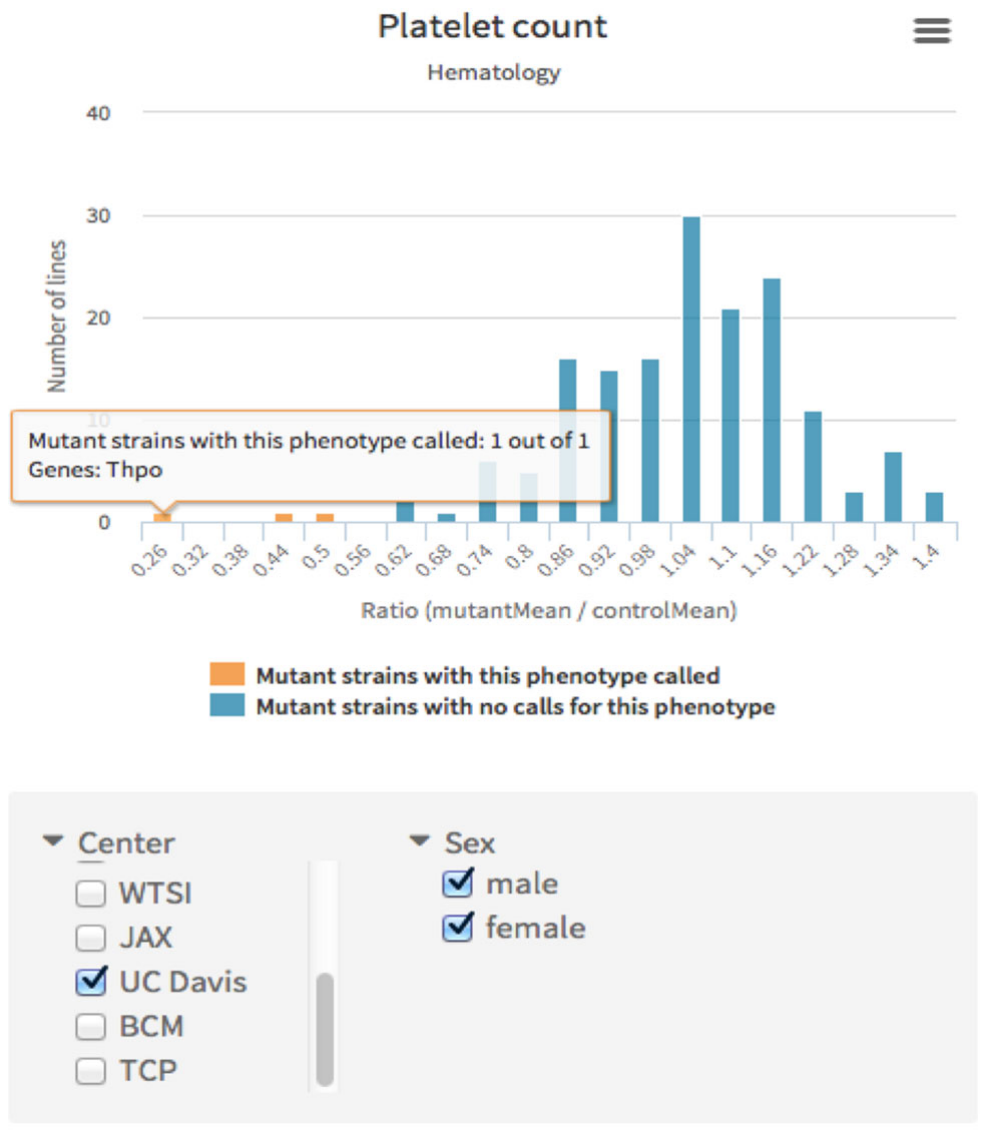
Platelet count obtained from the thrombocytopenia page. The histogram depicts the mean values for all tested strains and can be filtered by sex and by the phenotyping centre

Dedicated phenotype pages list a summary of all genes associated to a given phenotype, providing a valuable tool for researchers to discover new genes that share a phenotype with their genes of interest. Graphical and text summaries for a selected phenotype demonstrate the frequency by which a phenotype is associated to IMPC strains. Histograms provide distribution of mean values for each strain, and highlight which strains are phenodeviant. For example, a histogram of mean platelet counts of IMPC mice is available on the IMPC thrombocytopenia page which demonstrates that the *Thpo* knockout strain has the lowest mean platelet count of any strain characterised at UC-Davis (Fig. 5) (www.mousephenotype.org/data/phenotypes/MP:0003179).

### Incorporating images

IMPC’s data are image rich and the platform has evolved to support upload and display of images from both adult and embryo specimens. Adult mice images illuminate abnormal phenotype observations such as X-ray images showing abnormal curvature of the spine supporting abnormal vertebrae morphology assertions made by technicians at phenotype centres. Images of lacZ reporter expression can also inform about phenotype mechanism such as having positive lacZ staining in the epidermis of a mouse that has an abnormal coat phenotype. More than 100,000 adult phenotype images have been uploaded to date and the OMERO imaging management system is used to provide robust imaging archiving and dissemination. OMERO is an open source project focused on storage, presentation and analysis of biomedical images (Allan et al. 2012). OMERO supports conversion of proprietary imaging formats to web standard formats and provides online image viewers that have been implemented for the IMPC portal. MPI2 developers are working with OMERO developers to explore federation of images across IMPC centres and to directly associate critical phenotype metadata directly with images.

**Fig. 6:**
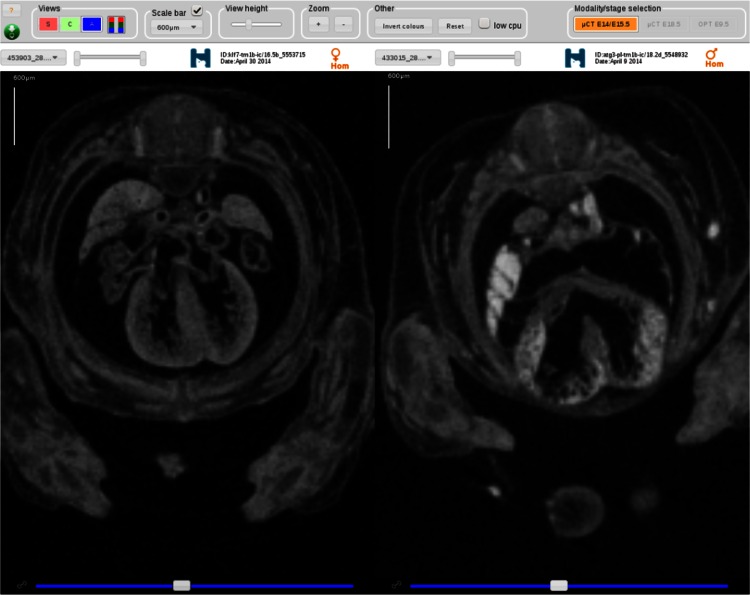
An example of micro-CT imaging of an E14.5 wild-type heart (*left*) and Atg3 null (*right*) visualised in IEV depicting a ventricular septal heart defect phenotype

Imaging is critical for assessing phenotypes in the one-third of knockout strains that are embryonic lethal. 3D imaging techniques such as OPT (Sharpe et al. 2002), micro-CT (Metscher 2009) and HREM (Weninger et al. 2014) are used to assess morphological differences in embryos at key developmental milestones (E9.5, E14.5–E15.5, E18.5). Three-dimensional image data require specialist tools for image capture, volume, display and analysis. MPI2 has developed the Harwell Automated Reconstruction Processor (HARP) tool that repackages 3D embryo reconstructions into a form that is easy to upload and process. HARP processes OPT and micro-CT data by cropping unnecessary black space, and compressing the file to make the transfer of large datasets more manageable (1–20 Gbs with compression). The 3D image data are displayed on the portal by the Internet Embryo Viewer (IEV) web tool. *IEV* includes features usually found in fully fledged desktop applications: image orientation changing, background colour swap (black to white), three different image orientation views (top, front, lateral), with possibility to toggle them on/off screen, zoom controls and more. The viewer is currently in testing (https://beta.mousephenotype.org/embryoviewer?gene_symbol=Atg3), and will be added to gene pages in a future release. Figure 6 shows an example visualisation for *Atg3* using IEV in the browser where a ventricular septal heart defect is visible. IEV offers functionality like orthogonal section views, slider bars allow movement through the image stacks, brightness and contrast controls and zoom.

MPI2 is also collaborating with the Mouse Imaging Centre (MICe; www.phenogenomics.ca/services/imaging.html) to automatically detect embryonic dysmorphologies using these 3D reconstructions. The MICe analysis pipeline automatically registers mutant and wild-type datasets together to determine if regions of significant dysmorphology (or absence) are evident (Wong et al. 2012, 2014). Automated detection of embryonic phenotypes is likely to increase sensitivity and conserve resources so MPI2 is piloting implementation of these analyses on IMPC resources.

### Integration of IMPC with other resources

All data found within the IMPC resource are associated to community-accepted vocabularies, including those developed by MGI. The IMPC uses the MP for all phenotype associations and the MGI’s Adult Mouse Anatomy Ontology (MA; Smith et al. 2014) for anatomical associations, and has worked with ontology developers to request new terms when sufficient ones do not suffice. This approach has allowed for rapid upload and reuse of IMPC data, which is particularly important as the volume of data being produced by the IMPC is rapidly increasing.

Users external to the IMPC also benefit from well-annotated data. Reciprocal links have been established between Ensembl (www.ensembl.org) and OMIM to allow users of these resources to benefit from IMPC phenotype associations. MGI imports and presents IMPC phenotypes as part of its efforts to associate phenotypes to mutant genotypes, while the MGI’s Gene Expression Database (GXD; www.informatics.jax.org/expression.shtml) is incorporating and displaying the lacZ reporter images that many IMPC centres are generating. The Illuminating the Druggable Genome project (IDG, www.commonfund.nih.gov/idg/) uses IMPC data as part of its efforts to collect knowledge about all druggable genes via an IMPC landing page (www.mousephenotype.org/data/secondaryproject/idg), and the Monarch Initiative (www.monarchinitiative.org) uses IMPC phenotypes in its effort to integrate phenotypes across all model organisms. One exciting application by Monarch is the use of IMPC phenotype data in the Exomiser tool (Robinson et al. 2014). This tool ranks candidate disease variants identified in exome analysis of rare disease patients based on model organisms that recapitulate the phenotypes of the patients. Exomiser is also in use by a number of consortia studying rare diseases such as the NIH Undiagnosed Diseases Program (www.genome.gov/27544402) and the Canadian Forge project (Beaulieu et al. 2014).

The IMPC also engages users by use of social media. A Twitter feed (@IMPC) informs followers about a wide range of IMPC activities such as new data releases, presentation and IMPC mouse of the week. This highlights mutant mice with interesting phenotypes such as a new disease models. An online training course and webinars provide users with information on how the data are generated and accessible (www.ebi.ac.uk/training/online/course/impc-using-mouse-phenotyping-portal). To date, the IMPC web portal has over 12,000 users per month with a steady trend of increased users.

## Conclusions

The IMPC’s informatics platform is constantly growing and evolving to provide swift and easy user access to our fast-growing dataset. We have ensured that phenotypic and translational outcomes are accessible through the portal by use of clinician friendly search terms and displays. We release the portal monthly, allowing us to respond to user feedback and the changing nature of our data. In the future, the platform will continue to develop dynamically as new data and uses become apparent. User-driven development will continue to be vital to this effort, making IMPC resources available for all in the most accessible manner possible.
